# Periodontitis as a Risk of Hospitalization and Death by SARS-CoV-2

**DOI:** 10.3389/ijph.2022.1605156

**Published:** 2022-08-31

**Authors:** Shipra Gupta, Ismo T. Räisänen, Timo Sorsa

**Affiliations:** ^1^ Oral Health Sciences Centre, Post Graduate Institute of Medical Education & Research, Chandigarh, India; ^2^ University of Helsinki and Helsinki University Hospital, Helsinki, Finland

**Keywords:** COVID-19, oral health, death, prevention, ventilation

Dear Editors,

We concur with the observations of Mauer et al. [1] addressing the comparative analysis of risks of hospitalization, clinical outcomes and death due to SARS-CoV-2 in patients hospitalized in Lombardy. Affordable precision/personalized medicine which combines the patients clinical data with lab-on-a-chip biomarkers, imaging and point-of-care diagnostics is the way forward if we want to dispense quality care to patients. Among real time monitoring of COVID-19 related risk diseases, it is pertinent to monitor oral and periodontal health, when we take into perspective the probability of poor gum health contributing to the COVID-19 related hospitalization and other adverse outcomes [2–4].

Sufficient evidence in literature underscores the importance of oral health, with emphasis on the prevention and treatment of periodontitis, linking it with many systemic diseases, and now with COVID-19 [3–5]. The same is suggested by the sample of 78 COVID-19 positive (delta variant) patients from India among whom patients with periodontitis were significantly more likely to need hospital admission, assisted ventilation or have COVID-19 related pneumonia than the periodontally healthy patients (*p* < 0.05; Table 1) [3]. Further studies are warranted to address the effect of oral and periodontal health on hospitalization and death due to other COVID-19 variants.

**TABLE 1:** Association between Periodontal disease status (Healthy, Gingivitis, Periodontitis) and Hospital admission, COVID-19 related pneumonia and assisted ventilation among 78 Indian COVID-19 positive patients (India, 2021).

	Healthy	Gingivitis	Periodontitis	*p*-value^a^
Home isolation	16^a^	12^a^	0^b^	<0.001
Hospital admission (Ward + ICU)	11^a^	9^a^	30^b^	
COVID-19 related pneumonia: Yes	3^a^	4^a,b^	14^b^	0.007
COVID-19 related pneumonia: No	24^a^	17^a,b^	16^b^	
Assisted ventilation required	3^a^	5^a^	21^b^	<0.001
Assisted ventilation not required	24^a^	16^a^	9^b^	

a Pearson Chi-Square (Exact Sig. 2-sided).

Each superscript letter denotes a subset of periodontal disease status groups whose column proportions do not differ significantly from each other measured by pairwise Z-tests (Bonferroni corrected) at the 0.05 level.
